# Responding to policy makers’ evaluation needs: combining experimental and quasi-experimental approaches to estimate the impact of performance based financing in Burkina Faso

**DOI:** 10.1186/s12913-019-4558-3

**Published:** 2019-10-22

**Authors:** Manuela De Allegri, Julia Lohmann, Aurélia Souares, Michael Hillebrecht, Saidou Hamadou, Hervé Hien, Ousmane Haidara, Paul Jacob Robyn

**Affiliations:** 1 Institute of Global Health, Medical Faculty, Heidelberg University, Germany; Im Neuenheimer Feld 130.3, 69120 Heidelberg, Germany; 2 The World Bank; Nouvelle Route Bastos B. P 1128, Yaoundé, Cameroon; 3 Centre MURAZ, 2054 Avenue Mamadou KONATE, 01 B.P. 390, Bobo-Dioulasso, 01 Burkina Faso; 4 The World Bank; Health, Nutrition, Population Global Practice, 1818 H Street, NW, Washington, DC 20433 USA

**Keywords:** Burkina Faso, Performance-based financing, Randomized controlled trial, Quasi-experiment, Difference-in-differences

## Abstract

**Background:**

The last two decades have seen a growing recognition of the need to expand the impact evaluation toolbox from an exclusive focus on randomized controlled trials to including quasi-experimental approaches. This appears to be particularly relevant when evaluation complex health interventions embedded in real-life settings often characterized by multiple research interests, limited researcher control, concurrently implemented policies and interventions, and other internal validity-threatening circumstances. To date, however, most studies described in the literature have employed either an exclusive experimental or an exclusive quasi-experimental approach.

**Methods:**

This paper presents the case of a study design exploiting the respective advantages of both approaches by combining experimental and quasi-experimental elements to evaluate the impact of a Performance-Based Financing (PBF) intervention in Burkina Faso. Specifically, the study employed a quasi-experimental design (pretest-posttest with comparison) with a nested experimental component (randomized controlled trial). A difference-in-differences approach was used as the main analytical strategy.

**Discussion:**

We aim to illustrate a way to reconcile scientific and pragmatic concerns to generate policy-relevant evidence on the intervention’s impact, which is methodologically rigorous in its identification strategy but also considerate of the context within which the intervention took place. In particular, we highlight how we formulated our research questions, ultimately leading our design choices, on the basis of the knowledge needs expressed by the policy and implementing stakeholders. We discuss methodological weaknesses of the design arising from contextual constraints and the accommodation of various interests, and how we worked ex-post to address them to the best extent possible to ensure maximal accuracy and credibility of our findings. We hope that our case may be inspirational for other researchers wishing to undertake research in settings where field circumstances do not appear to be ideal for an impact evaluation.

**Trial registration:**

Registered with RIDIE (RIDIE-STUDY-ID-54412a964bce8) on 10/17/2014.

## Background

In the year 2000, the Medical Research Council (MRC) issued guidelines to orient researchers interested in the evaluation of complex health interventions. The abridged version of the MRC guidelines published by Campbell and colleagues [1] drew wide attention to the need to identify an adequate framework for the design and evaluation of complex interventions aimed at improving health. They defined complex interventions as health interventions that rely on multiple components not fully under the researcher’s control. While undoubtedly deserving the merit of first drawing attention to the special circumstances under which complex health interventions are undertaken in comparison to standard clinical trials, the early work by Campbell and colleagues reinforced the central role of randomized trials in evaluation research rather that to encourage a broadening of the methodological toolbox.

In response to the widespread debate spurred by their publication [2–4], the initial MRC guidelines were substantially revised in 2008 [5, 6], with the explicit objective of paying closer attention to the relationship between implementation processes and outcome evaluation. The revised guidelines included specific guidance to researchers when choosing between randomized and non-randomized designs and drew attention to the need to invest in complementary process evaluations while assessing an intervention’s impact.

The development of the MRC guidelines over time reflects an overall evolution in the literature. Although randomized controlled trials continue to play a key role in impact evaluation [7–9], researchers today acknowledge the need to think beyond the narrow framework of randomization and have come to rely increasingly more often on quasi-experiments when called to evaluate the impact of complex health interventions [10–14].

It is surprising, however, that little attention has so far been paid to how experiments and quasi-experiments can be combined within a single study design. The existing literature presents experiments and quasi-experiments as alternative means of achieving a given evaluation objective rather than viewing the two as complementary approaches to be combined when in need to evaluate complex health interventions [15–18]. Combining the two appears to be particularly relevant considering that complex health interventions are not implemented in a vacuum, but occur within real-life settings often characterized by limited researcher control, concurrently implemented policies and interventions, and other internal validity-threatening circumstances.

This paper presents the case of a study design combining experimental and quasi-experimental elements to evaluate the impact of a Performance-Based Financing (PBF) intervention in Burkina Faso. In line with the work by Habicht et al. [19], we use the case to elucidate how the design decisions our research team (Institute of Global Health, Heidelberg University, Germany, and Centre MURAZ, Burkina Faso) made were guided by the knowledge needs of the policy makers on behalf of whom we conducted the impact evaluation between 2013 and 2017. Specifically, we illustrate how experimental and quasi-experimental elements were combined within a single evaluation design to answer different research questions while respecting the context within which the implementation of the intervention took place. We describe the trade-offs that arose when trying to accommodate both the multiple research interests and the realities of the context and the intervention itself, as well as the analytical techniques we applied to address them. In doing so, we intend our case to serve as an illustration for other researchers planning to conduct rigorous impact evaluations in settings where circumstances do not appear ideal and/or where multiple research interests need to be reconciled within a single study.

## Methods

### Study setting

In order to understand the intervention and the design decisions we made within the framework of our impact evaluation, we first provide some background information on the country and its health system.

Burkina Faso is a landlocked country located in West Africa, with a population of 18.5 million. At the time the study was planned, the country’s GDP per capita stood at USD 1560 (2013, adjusted for purchasing power parity) placing it among the poorest countries in the world [20]. The 2014 Human Development Index ranked Burkina Faso 185 out of 188 countries [21].

In spite of substantial improvements over the course of the last few years, health indicators still largely lag behind regional averages. Life expectancy is at 58 years. Maternal and under-five mortality are estimated at 371/100,000 [22] and 102/1000 [23], respectively. Malaria, acute respiratory infections, and diarrhea still account for the largest proportion of child mortality, often coupled with an underlying situation of malnutrition, with nearly 40% of all children being classified as stunted.

Health service delivery is organized in a three-tier system, with primary facilities (Centre de Santé et Promotion Sociale - CSPS) located in rural areas; district hospitals located in each district capital; and regional and national referral hospitals located in the regional capitals and in the national capital Ouagadougou [24]. Public facilities provide the vast majority of health services [25].

The health sector suffers from a generalized lack of resources. In 2013, total per capita health expenditure was estimated at 6.4% of GDP, equivalent to Purchasing Power Parity USD 109. Government expenditure amounted to 58% of total health expenditure, including contributions by development partners being estimated at 23% of this total. Private health expenditure is substantial as user charges continue to be applied across a variety of essential healthcare services, with more than 80% of all private expenditure on health not being channeled through pre-paid and pooled mechanisms [26, 27].

The poor health outcomes described above are, to a large extent, the result of poor access to services, with people largely under-utilizing the care they need. The literature has consistently reported that geographical barriers, due to scarcity of health facilities, and financial barriers, due to user charges, continue to hamper access to healthcare services [28–33].

### Intervention design

The PBF program at the core of our impact evaluation rests on the experience and knowledge acquired during the implementation of a pre-pilot PBF intervention, managed by the Ministry of Health (MoH) with financial and technical assistance from the World Bank in the period 2011–2013 in three districts (Titao, Leo, and Boulsa). Within the framework of this early PBF intervention, health facilities and the MoH entered a contractual agreement whereby the MoH would reward the provision of a defined service package according to a case-based payment modality, adjusted for quality of service provision and following verification. An independent evaluation detected a positive effect of the intervention across maternal healthcare services [34]. Program data showed improvements in the quality of services provided [35].

In light of the pre-pilot experiences, the MoH, again with financial and technical assistance from the World Bank, decided to scale up the PBF intervention to an additional 12 districts in early 2014. In doing so, it was decided to test the combination of PBF with other policies under consideration for scale-up or integration into the national health system, notably community-based health insurance (CBHI) and user fee exemption for the ultra-poor [36, 37].

To understand this decision, it is important to locate the PBF program in Burkina Faso within the broader context of PBF programs supported by the World Bank over the course of the past decade through the Health Results Innovation Trust Fund (HRITF), including impact evaluations in over 20 countries, of which the majority in Sub-Saharan Africa. Emerging preliminary findings across the existing pilots suggested PBF’s potential to improve service provision, but pointed at PBF’s inability to reduce inequality in service access, specifically when implemented exclusively as a supply-side intervention [38].

It is also in the light of these considerations that the MoH and its development partners opted to implement PBF in conjunction with a series of equity measures, aimed at maximizing the potential of PBF to act as a catalyst for equity changes. It is at this point, building on the knowledge generated in other settings and looking at the specific need to address equity gaps in the country, that the World Bank realized the potential to use the case of Burkina Faso to test novel financing, purchasing and targeting mechanisms, combining elements of supply and demand side interventions into a single program. It is also at this point that knowledge generation was conceptualized as an intrinsic component of the PBF program implementation and that the decision to contract an independent academic institution to carry out the impact evaluation was made. It ought to be noted, as described in detail in the sections that follow, that the research team was not involved in key intervention design decisions (i.e. selection of intervention districts, test of equity measures), but did contribute to shaping the evaluation design as well as to elaborating the intervention design (i.e. exact design of the equity measures).

Similarly to the pre-pilot, the primary objective of the PBF program was to improve the utilization and quality of maternal and child health (MCH) services, in particular among vulnerable populations such as the ultra-poor. Effectively, however, the PBF benefit package was very comprehensive and comprised a broad range of primary- and secondary-level care services beyond MCH, including also general adult curative consultations and HIV and tuberculosis services [39]. To address the abovementioned equity concerns, the MoH decided to implement four different models of PBF, three of which included special provisions to improve access to care for the ultra-poor by explicitly alleviating the financial burden imposed by user charges.

In detail, the following four PBF models were implemented (Table 1):

**Table 1:** “Intervention design”

T1: PBF	T2: PBF + systematic targeting and subsidization for the poor
T3: PBF + systematic targeting and subsidization for the poor + additional incentives for consultations of the poor	T4: PBF + CBHI + systematic targeting and subsidization for the poor

*PBF1*: Standard PBF. PBF contracts between the MoH and the health facilities defined the services purchased by PBF (quantity), quality targets, and payment modalities. External reviewers assessed facility reports on quantity indicators on a monthly basis. Based on these verified results, contracted facilities received case-based payments for the services delivered, in addition to all preexisting financing. PBF unit prices were calculated a priori by the implementation team, on the basis of the relative cost and frequency of the services provided. Service quality was assessed with comprehensive quality checklists, verified on a quarterly basis by the District Health Management Teams. Facilities received an additional bonus calculated on the basis of quantity outcomes and service quality if they achieve a quality score of initially at least 50%, later 60%. PBF payments were intended to supplement other facility revenue in the complex pre-existing mixed payment system [40], to fund expenditure, increase savings, and allow bonus payments to staff. Facilities were initially completely autonomous in their spending decisions. In 2017, ceilings as to how much could be disbursed to staff were introduced in response to undesirable practices in some facilities. Facilities were further provided with criteria for the distribution of bonuses among staff, including cadre, position, seniority, work time, and performance. PBF1 did not include any specific provision to facilitate access to care for the ultra-poor.

*PBF2*: Standard PBF + systematic targeting and health service subsidization for the ultra-poor. PBF2 operated according to the same contractual model as PBF1, but combined it with specific provisions to facilitate access to care for extremely vulnerable individuals residing in the health facility catchment area. These provisions included:

- A systematic targeting of the ultra-poor (‘indigents’) was implemented using a community targeting approach [41], facilitated by SERSAP (Société d’Etudes et de Recherche en Santé Publique). The aim was to identify up to 20% of individuals residing in the health facility catchment area and provide them with proof of indigent status to allow them to access all services included in the PBF benefit package free of charge.
- Unit prices for services delivered to the targeted ultra-poor were adjusted to compensate for the loss of revenues that health facilities incurred by not charging user fees to this specific sub-population of users. The additional payments were exclusive to services for which direct user charges existed (e.g. curative consultations, delivery services, family planning), but excluded services which were already provided free of charge (e.g. HIV and tuberculosis testing and treatment, vaccinations).

*PBF3*: Standard PBF + systematic targeting and subsidization for the ultra-poor + provider incentive to offer services to the ultra-poor. PBF3 operated the same PBF contracts as PBF1 and PBF2 and included the same targeting procedures and provisions to care for the ultra-poor as PBF2. The core difference relates to the unit prices applied in PBF3, in that services provided to the ultra-poor were reimbursed at a higher rate than in PBF2 (initially at around 150% of the PBF2 prices, reduced to an average value of 115% in 2016, with variation across services). The idea was to compensate for loss of income from user fees, while at the same time offering providers an additional financial incentive to attract and provide services to the ultra-poor. As in PBF2, these higher reimbursement rates only pertained to services normally offered against payment of direct user charges at point of use.

*PBF4*: Traditional PBF + Community-based health insurance (CBHI), including targeting and subsidization for the ultra-poor. In this case, PBF1, applying the same contractual model as described above, was introduced in parallel to CBHI. The insurance scheme was rolled out with support from the NGO ASMADE, which developed a scheme based on the model that the government had envisioned for a future universal health insurance model (Régime d’Assurance Maladie Universelle) [36]. ASMADE was selected as implementing agency given its prior experience with insurance implementation. Insurance was offered to the entire population at a yearly premium of 3900 FCFA (~ 7 USD) per person. Targeting took place following procedures similar to the ones used in PBF2 and PBF3 areas and the insurance premium for the ultra-poor was fully subsidized by the program. The insurance benefit package included a wide range of primary and secondary healthcare services. Payments to providers were made by both the insurance (in place of user charges) and by the PBF program, as case-based rewards as in PBF1.

Across the four PBF models, adjustments to the quantity case-based payments were made according to the remoteness of the catchment population, staffing levels, and distance from the district capital, so that remote and disadvantaged facilities received higher case-based payments than easily accessible and better-equipped facilities. This approach resulted in nine different possible prices for the services incentivized by PBF, beyond the adjustments made in PBF2 and PBF3 for services provided to the targeted ultra-poor.

The PBF program was rolled out in six regions (Centre Nord, Centre Ouest, Nord, Sud Ouest, Boucle du Mouhoun, and Centre Est), purposely selected by the MoH and its development partners as having health indicators below the national median at the onset of the intervention [42]. Within each region, the MoH purposely selected two districts to receive PBF on the basis of particularly poor outcomes on four key indicators: (i) contraceptive prevalence rate; (ii) assisted deliveries; (iii) antenatal consultations; and (iv) post-natal consultations.

### Study design

Identifying an adequate study design for the impact evaluation required an engagement of the main stakeholders (including MoH, the World Bank, and the independent impact evaluation research team) in an iterative discussion in which policy interests were appraised against both scientific considerations and pragmatic implementation concerns. The primary interest of the technical partners, particularly the World Bank, was to test the added benefit of moving from the standard PBF model to one that combined PBF with specific equity measures. The MoH, on the other hand, was primarily interested in identifying the overall impact of introducing PBF to generate evidence to inform future decisions on health financing in the country.

The most straightforward design to accommodate both interests would have been a random allocation of all health facilities to one of the four different PBF intervention models or to a control group (i.e. status quo). This design was judged unfeasible from the policy makers’ perspective for four reasons. First, policy makers feared that randomization to either PBF or control within single districts was unfeasible since districts represent the primary operational units in the Burkinabè decentralized healthcare system and could have therefore not applied different purchasing strategies across the facilities they controlled. Second, policy makers feared that randomization to PBF and control within a single district might lead to conflict since the intervention would be highly visible and people from control groups could feel left behind. In addition, results from similarly complex health interventions with full-scale randomization report on the presence of spillover effects, due to consumers’ mobility across facilities, to shared management structures at the district level, and to intra-district competition, posing a challenge to the internal validity of the evaluations design [43, 44]. Third, the implementation of a CBHI scheme appeared to be too complex an intervention to be allocated randomly across facilities in twelve districts. The level of know-how necessary to facilitate insurance implementation was absent in most districts, hence from the very onset of the discussions, the government made clear its intention to test the insurance model in conjunction with PBF exclusively in one region, Boucle du Mouhoun, where prior experience with insurance implementation was present [45, 46]. Fourth, it became apparent that the implementation of the targeting component would be quite costly and that funds would not cover its implementation across all twelve districts.

**Fig. 1:**
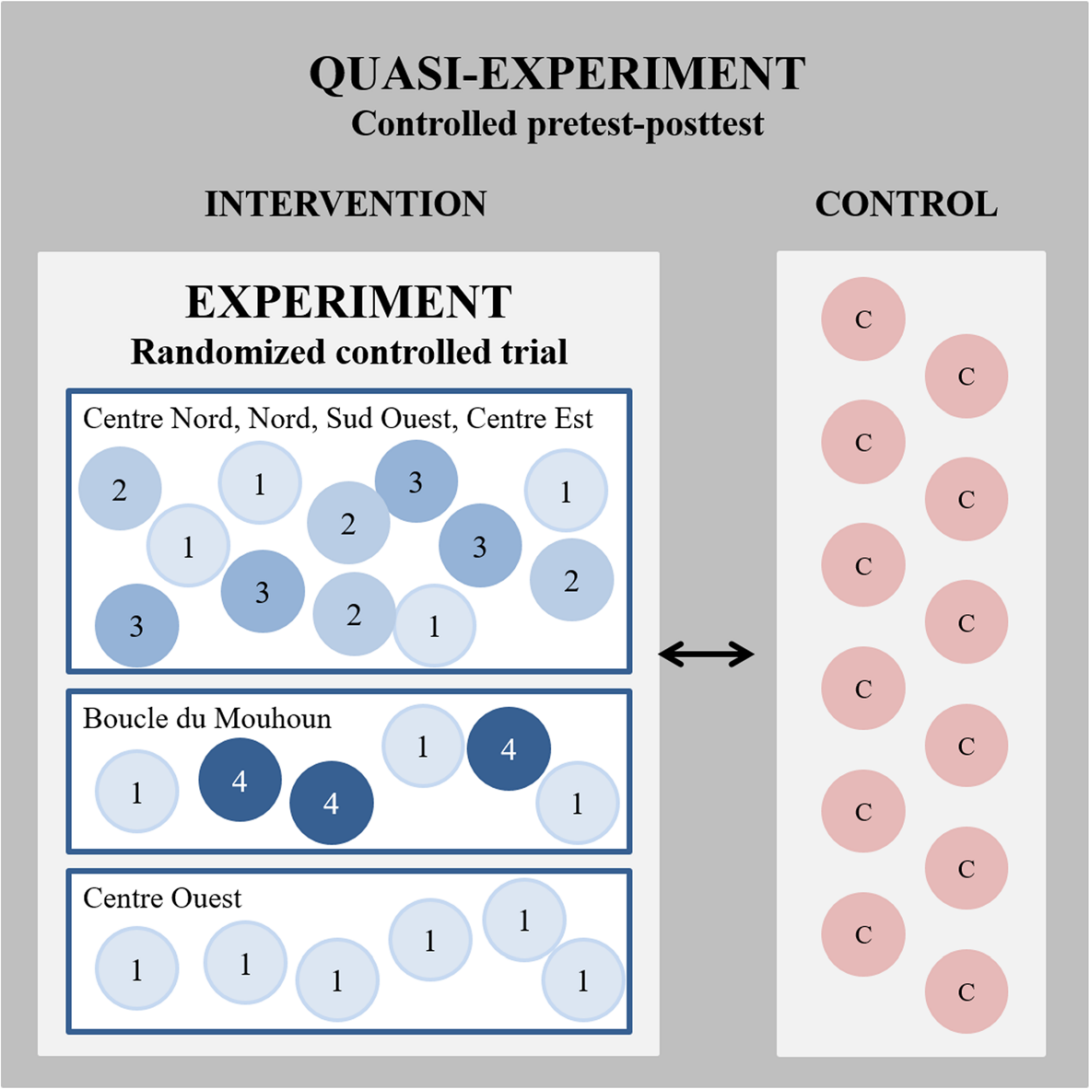
“Study design”

Hence, the research team and the policy stakeholders agreed to employ a quasi-experimental design with a nested experimental component (Fig. 1). In practice, this meant that within each region, two additional districts, judged by policy stakeholders to be comparable in terms of health indicators and health system structures, were selected as controls. The twelve control districts received no PBF intervention at all. Within the twelve intervention districts, the four PBF packages described earlier were implemented as follows:

- In eight districts, PBF1, PBF2, and PBF3 intervention packages were randomly allocated to health facilities and their catchment areas;
- In two districts, PBF1 and PBF4 intervention packages were randomly allocated to health facilities and their catchment areas;
- In two districts, only the PBF1 intervention package was implemented for budgetary reasons.

Within the concerned intervention districts, facilities were allocated across the different PBF models in ‘randomization ceremonies’, attended by all health facility in-charges, district health managers, and other important district and regional stakeholders to maximize transparency. During the ceremonies, after a brief introduction of the program, health facility in-charges then took turns drawing facility names from a box containing all health facility names in the respective district. Starting with a predefined PBF model, facilities were then assigned in the order in which they were drawn from the box (i.e. 1st facility: PBF1, 2nd facility: PBF2, 3rd facility: PBF3, 4th facility: PBF1, etc.). As referral facilities to all health centers in the districts, the twelve district hospitals as well as the few regional hospitals were not randomized, but rather assigned to the PBF2 intervention package and therefore reimbursed for user fee-free treatment of targeted poor. It should be noted that although secondary-level facilities were included in the intervention to ensure continuity of care, the study focus was clearly on primary-level facilities.

This design accommodated the concerns related to implementation complexity raised by policy makers, while still granting the possibility to answer their research questions. As explained in greater detail later, the quasi-experimental element of the design was used to assess the overall impact of the PBF program (irrespective of the specific intervention package) vis à vis status quo health service provision. The experimental element of the design was used to assess the specific added benefit of introducing equity measures (as in PBF2, PBF3, and PBF4) alongside the implementation of standard PBF (PBF1).

It should be noted that the decision not to randomize across all covered health facilities came at the cost of having the study design’s statistical power to detect the impact of PBF compared to status quo reduced, due to a low number of clusters (defined as the districts for the quasi-experimental component) [47]. This concern was discussed among stakeholders already during the design phase. An expansion of the pilot intervention to a larger number of clusters, however, was not feasible due to financial and pragmatic constraints. Researchers and policy stakeholders therefore accepted this limitation, also in light of the fact that the primary research interest was related to the experimental component.

### Study objective and research questions

The overall objective of the impact evaluation was to assess the impact of the PBF program on health service utilization and quality of service delivery across a wide range of targeted services. As described above, the specific focus of the impact evaluation, when compared to existing studies [48–51], was on estimating the added benefit of combining PBF with equity measures (i.e. either the ultra-poor targeting and health service subsidization or the CBHI).

The specific research questions fitting the abovementioned objectives were:

1. What is the effect of the PBF program (irrespective of specific design model) on selected service utilization and quality of service delivery indicators compared to status quo service provision?
2. What is the effect of the specific PBF models (PBF1, PBF2, PBF3, PBF4) on selected service utilization and quality indicators compared to status quo service provision?
3. What is the added benefit of implementing PBF2, PBF3, and PBF4 compared to the standard PBF1 on selected service utilization and quality indicators?

To address the equity implications embedded in the program, for each of the abovementioned three primary research questions, we further asked:

4. What is the effect of PBF on selected service utilization and quality of service delivery indicators among the very poor? What is the added benefit of implementing PBF2, PBF3, and PBF4 beyond the standard PBF1 among the very poor?

### Theory of change

**Fig. 2:**
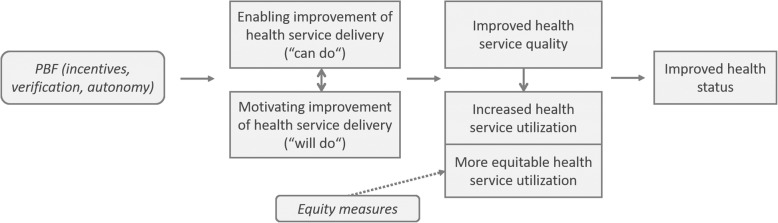
“PBF theory of change”

In line with the existing conceptual literature on PBF [52–54], we postulated that the standard PBF intervention (i.e. the performance contracted implemented across all four PBF intervention packages) would affect both quantity and quality of service provision (Fig. 2). Specifically, we expected the combination of increased revenues due to performance rewards, enhanced managerial autonomy fostered by PBF, and intensified supervision on service provision to motivate healthcare providers to actively engage to increase health service provision (“will do”), while at the same time empowering them with the financial and managerial means necessary to improve quality of service delivery (“can do”). In turn, we expected improved quality of service delivery to encourage communities to seek healthcare more promptly (in spite of the fact that the Burkinabè health system still largely relied on direct user payments at point of use throughout the duration of the study), further increasing quantity of service delivery. We expected the standard PBF intervention to be equity-neutral, i.e. not to favor nor disfavor any specific socio-economic segment of society.

We expected the combination of the standard PBF1 and equity measure as applied by PBF2 and PBF3 to stimulate increases in quantity of service provision both overall and specifically for the ultra-poor. Across both the PBF2 and the PBF3 intervention arms, we expected the targeted exemptions to motivate the ultra-poor to seek healthcare more promptly and we expected this increased health service utilization to narrow existing gaps in the quantity of service delivery provided across socio-economic groups. We expected the socio-economic gap in quantity of service delivery to decrease more drastically in the PBF3 intervention arm, given the additional financial incentive offered to providers to actively reach out to the ultra-poor. In addition, given that services were purchased at a higher value than their estimated cost, we expected PBF3 facilities to increase their revenues substantially more than PBF1 and PBF2 facilities and in turn, we expected these additional revenues to be deployed towards infrastructural investments, further contributing to quality improvements in PBF3 facilities.

Similar to what we described above in relation to PBF2 and PBF3, we postulated that the PBF4 intervention package would stimulate larger increases in the quantity of service delivery than the standard PBF1. We expected that increased health service utilization linked to the removal of financial barriers at point of use (thanks to insurance) would result in higher quantities of service delivery [55–59]. We also expected to observe larger improvements in the quality of service delivery in PBF4 facilities compared to PBF1 facilities, because we expected the CBHI scheme to act as an additional independent purchaser, exercising pressure on healthcare providers and hence acting as an additional stimulus for quality improvement. Given that targeting was implemented in a similar fashion across PBF2, PBF3, and PBF4 catchment areas, we expected to observe similar equity impacts, although somewhat more pronounced in PBF3 due to the additional provider incentive to reach out to the targeted ultra-poor.

As addressed earlier when describing the intervention design, the list of incentivized services was identical across all four intervention models and it was very comprehensive, effectively covering almost the entirety of the essential package of services at primary and secondary levels. Hence, we did not expect healthcare providers in PBF1 and PBF4 to focus their efforts on specific services, simply because the payment structure was not set in a way as to offer any incentive to privilege some services above others. In the PBF2 and PBF3 intervention arm, however, the additional payments to compensate for loss of revenues from user fees and the additional financial incentive to treat the ultra-poor in PBF3 were exclusively tied to services normally offered against payment of direct user charges at point of use. Consequently, we expected that, when treating the very poor, providers would have an incentive to provide services for which they receive both the standard PBF payment and the additional compensation related to the attended loss of income from user fees/additional financial incentive (such as curative consultations) rather than services for which they received only the standard PBF payment (such as vaccination or antenatal care (ANC)). Therefore, in PBF2 and PBF3 facilities, we expected to observe an overall greater increase in case volume as well as a more remarkable equity improvement specifically for these services for which additional compensation was offered.

Across the four PBF models, we expected increases in the quantity and quality of service provision to result in improved health status, especially among women and children, given that most indicators targeted MCH services. We did not expect the intervention to produce measurable changes in mortality patterns over its short implementation period, but we did expect the improved health service delivery resulting from PBF to be able to produce changes in simpler health status indicators, such as those related to anemia and acute malnutrition.

### Outcome indicators

Table 2 contains the set of main outcome indicators selected for our impact evaluation. In their selection, we took into account the above-described theory of change, including indicators at the different levels, the list of incentivized services, national and international standards, as well as data availability, quality and baseline values. In alignment with the focus of the study, the set of main outcome indicators pertains exclusively to the primary level of care.

**Table 2:** Main impact evaluation outcome indicator set

INDICATOR		DATA SOURCE
Indicators pertaining to human resources		
1	Proportion of staff having been evaluated for their performance in last year	Health worker survey
2	Health workers’ perceived individual agency	
3	Health workers’ satisfaction with the physical work environment	
4	Health workers’ satisfaction with their compensation	
5	Health workers’ satisfaction with management and supervision	
6	Health workers’ intrinsic motivation	
Indicators pertaining to health service quality		
7	Proportion of facilities with permanent availability of power and safe water in the last 7 days	Health facility assessment
8	Proportion of facilities with at least one unit of 23 essential drugs in stock	
9	Proportion of facilities with availability and functionality of key equipment for consultations of children under 5	
10	Proportion of facilities with availability and functionality of key equipment for ANC	
11	Proportion of facilities with availability and functionality of key equipment for delivery	
12	Proportion of observed ANC cases having received three key routine ANC services	Direct observation
13	Proportion of observed ANC cases having received patient education on three key elements	
14	Proportion of children observed in curative consultations having been assessed for all IMCI danger signs	Direct observation
15	Proportion of children observed in curative consultations having been assessed for common childhood illness symptoms according to IMCI	
16a	Proportion of ANC clients perceiving adequate quality of care on seven key elements	Exit interview
16b	Proportion of U5 consultation clients perceiving adequate quality of care on seven key elements	Exit interview
16c	Proportion of curative consultation clients aged 5 or older perceiving adequate quality of care on seven key elements	Exit interview
Indicators pertaining to the utilization of reproductive health care services		
17	Proportion of recently pregnant women with at least four ANC visits	Household survey
18	Proportion of recently pregnant women with an ANC visit within first four months of pregnancy	
19	Proportion of recently pregnant women having received at least 2 doses of tetanus vaccine during pregnancy	
20	Proportion of recently pregnant women having been offered HIV testing during pregnancy	
21	Number of HIV-positive mothers who have completed prophylactic ARV treatment	HMIS
22	Proportion of recently pregnant women who have delivered in a formal health facility	Household survey
23	Proportion of recently pregnant women with at least one PNC visit within 6 weeks after delivery	
24	Proportion of recently pregnant women with at least three PNC visits within 6 weeks after delivery	
25	Proportion of non-pregnant women aged 15–49 who use modern family planning methods	
Indicators pertaining to the utilization of preventive child health services		
26	Proportion of children aged 12–23 months who are fully immunized (primary data)	Household survey
27	Proportion of children aged 0–11 months who have participated in growth monitoring in last 6 months (primary data)	
28	Proportion of children aged 12–23 months who have participated in growth monitoring in last 6 months	
Indicators pertaining to the utilization of curative health care services		
29	Number of patients under age 5 having sought curative services	HMIS
30	Number of patients age 5 or older having sought curative services	
Indicators pertaining to population health status		
31	Proportion of children aged 0–59 months who are severely stunted	Household survey
32	Proportion of children aged 0–59 months with severe acute malnutrition	
33	Proportion of children aged 6–59 months with anemia	
34	Proportion of women aged 15–49 years with anemia	

An equity analysis was possible for all health care utilization and health status indicators for which data stems from the household survey, but not for indicators based on HMIS data. No equity analysis was possible for quality of care or human resources indicators

### Data sources and data collection tools

The impact evaluation relied on two main sources of data: i. a household survey, conducted at baseline (October 2013–March 2014) and endline (April–June 2017); ii. a facility-based survey, also conducted at baseline and endline, including different data collection tools: a health facility assessment, a health worker survey, direct provider-patient observations (ANC and curative services for children under the age of 5 (U5)), and patient exit interviews (ANC, U5 curative services, and curative services to patients aged five or older). In addition, we used data from the routine health management information system (HMIS) to triangulate results obtained with primary data, and to estimate impact on indicators for which our sampling strategy did not generate a sufficiently large sample, namely health care utilization for acute illness and ART for prevention of mother-to-child transmission. We pooled the respective six months of routine data before the primary baseline and endline data collections and treated the resulting data points the same way as the primary data in the analyses described in the following.

To collect primary data, we used a slightly revised version of the data collection tool set included in the HRITF impact evaluation toolkit [60] tailored to the needs of this specific impact evaluation and to the Burkinabé context. Table 3 illustrates the content of each data collection tool employed in our impact evaluation.

**Table 3:** Data collection tools and sources

Data collection tool	Data source	Description of Data
Household survey	Household head or representative	Household demographic and socio-economic profile, deaths in the 10 prior years
Household survey	All household members	Chronic conditions and health service use; health service use for acute conditions in the 4 weeks preceding the survey (incl. expenditure)
Household survey	Currently pregnant women and women who have ended a pregnancy in the 24 prior months	Pregnancy and birth history, use of reproductive health services (incl. costs), perceptions of health service quality
Household survey	Children under 5	Immunization status, use of growth monitoring services
Household survey	Women of reproductive age, children under 5	Rapid diagnostic tests for malaria & anemia, height and weight measurements
Facility assessment	Facility in-charge	Service availability, administrative information (including finances), staffing, infrastructure, availability of infrastructure, equipment, drugs, and consumables
Health worker survey	Skilled health care personnel	Training, clinical knowledge, compensation, supervision, motivation, satisfaction, perceived working conditions
Direct observation	Antenatal care consultations, curative consultations for children under 5	Case management, treatment and counseling provided
Patient exit interview	Patients exiting from antenatal care consultations, curative consultations for children under 5, and curative consultations for patients aged 5 and older	Patient’s (or caretaker’s) perception of quality of care and satisfaction, socio-economic information
HMIS	Health facilities	Patient counts per service category from the routine health management information system

Data collection teams spent one day at each sampled health facility and village, collecting data partially on paper (facility-based survey) and partially electronically (household survey) at baseline, and fully electronically at endline. In order to ensure efficient data collection, field supervisors travelled ahead of their teams to observe social protocols, finalize sampling before arrival of the data collection team, and ensure availability of interview partners. An independent quality assurance control of the endline data collection process was commissioned to the *Institut de Recherche en Sciences de la Santé* (IRSS), Burkina Faso.

### Sample and data collection procedures

Our sampling strategy seeked to cover all health facilities and their attached catchment areas included in the intervention at baseline. Ideally, we would have liked panel data for all indicators, with the same units measured at baseline and endline, to ensure a maximally robust impact estimation. However, this was only partially possible, as explained below.

#### Health facility survey

The health facility survey was conducted in all public and private primary- and secondary-level health facilities in the intervention districts providing essential reproductive and child care services, as well as a random sample of health facilities in the control districts for an intervention-control facility ratio of approximately 3:1. This amounted to a total of 537 primary- and 24 secondary-level facilities in the 24 intervention and control districts surveyed at both baseline and at endline. The health facility sample was therefore a fully balanced panel. Health facilities newly opened in intervention districts between baseline and endline were included in the PBF intervention, but not in the impact evaluation sample.

*Health facility assessments* were conducted in all health facilities included in the sample. In primary care facilities, the *health worker survey* was applied to all clinical skilled healthcare staff available on the day of the visit. Random sampling from all staff on payroll or a census was not feasible for reasons of time and budget. Due to high staff turnover and for the same budgetary and time reasons, it was not possible to systematically reinterview baseline respondents at endline. At secondary facilities, the health worker survey was administered to a random sample of three health workers with maternal and child health service delivery responsibilities. At all facilities where ANC services were provided on the day of the interviewer team visit, five *direct provider-patient observations of ANC consultations* were performed. Specifically, interviewers were instructed to observe the first five consultations as random sampling was not feasible for pragmatic reasons. Similarly, five *direct provider-patient observations for U5 curative consultations presenting with a new condition* were performed at all health facilities included in the sample. We further performed exit interviews with the observed patients (or their caregivers) for both service categories (ANC, U5 curative services), as well as with five curative consultation patients aged five and above at each sampled health facility. As observations and exit interviews depended on the patients naturally presenting on the day of data collection, the construction of a panel at this level was not possible.

#### Household survey

One village was randomly selected from the catchment area of each of the 523 public primary health care facilities included in the intervention. Since the secondary and few private facilities do not have an own catchment area, we could not sample a specific village at their level. The same villages were visited at baseline and endline, resulting in a fully balanced panel at the village level. In each selected village, at baseline, we randomly selected 15 households among all households with at least one currently pregnant woman or at least one woman having ended a pregnancy (irrespective of outcome) within the prior 24 months. Eligible households were identified through a preliminary comprehensive listing of all households in the selected villages, conducted by the same field enumerators who later administered the survey. This sampling criterion was chosen to allow obtaining sufficiently large sample sizes for the key indicators of interest (i.e. utilization and quality of maternal and child health care services) at much lower cost than with a fully representative population sample, which would have exceeded the budget available. We are aware that such a sample, which is not fully representative of the population in the concerned districts, limits our ability to approach research questions not directly pertaining to MCH.

At endline, we organized data collection at the household level in such a way as to create, at least to the extent possible, a partial panel. Hence, we first returned to baseline households at endline. If they still fulfilled the abovementioned sampling criterion, they were included in the endline survey. If not, they were replaced with the nearest household meeting the above-outlined eligibility criteria. Our expectation was that a relatively large share of baseline households would still be eligible at endline, given high fertility rates in Burkina Faso and a three-year lag period between baseline and endline. However, we could identify and include in the endline household sample only 53% of baseline households, while 38% were no longer eligible, 5% could not be traced again and 4% were still eligible, but refused to participate again. Additional analyses showed that while the partial panel introduced some selection bias to our sample (slightly higher fertility in panel households), the bias was constant across study groups, and no other observable systematic differences between panel and non-panel households and individuals were apparent.

### Analytical approach

While keeping alignment with the overall HRITF analytical framework and strategy, our analytical approach had to accommodate the specific nature of our study design, research questions, and data structure. In line with what was described earlier in this manuscript, we nested an experimental component within a broader quasi-experimental design to deliberately address the knowledge needs raised by the different policy stakeholders.

As we had a balanced panel only at the health facility/village level, but not at the health worker, patient, household, and household member levels, where the majority of key indicators was measured, we could not conduct observation-level panel analyses, and therefore treated the baseline and endline samples as repeated cross-sections. Important to note is that this is not necessarily a specific weakness of our study, but rather the norm in studies pertaining to health system interventions [61].

We employed a Difference-in-Differences (DID) approach to identify the impact of PBF compared to status quo and the added benefit of the equity measures in PBF2, PBF3, and PBF4 compared to the standard PBF1. In DID, the intervention effect is estimated as the difference between the baseline-endline change in the intervention group and the control group. DID thereby isolates the intervention effect from baseline differences between the study groups, as well as from secular trends over time [47]. In contrast to our nested experiment, where impact estimates can be identified through a simple difference approach, causal inference in our quasi-experimental setting makes a DID approach necessary. In our specific case, we indeed observed significant differences between the intervention and control group on certain indicators at baseline, which are likely driven by the purposive way in which intervention and control districts were selected. We also observed secular positive and negative trends on many indicators, in light of general developments in the country and diverse national efforts and policies, most notably the exemption policy targeting pregnant and lactating women and children under five (the ‘politique de gratuité’) introduced nationally in June 2016.

DID relies on two main assumptions: 1) the ‘parallel trend assumption’ that intervention and control units of observations would have in fact developed in the same manner in the absence of the treatment; and 2) the ‘stable unit treatment value assumption’ that each unit of observation was clearly either exposed to the treatment or not (i.e. no spillover) and that treatment was uniform across all units assigned to it (i.e. no contamination) [47]. To the extent possible, we aimed to validate these two assumptions with additionally collected information and data. In regards to 1), using HMIS, we found uniformity of pre-intervention trends among the different study groups on the indicators pertaining to health care utilization, strengthening our confidence in the control districts as an appropriate approximation of the counterfactual. In regards to 2), we systematically collected information on other interventions on-going and newly introduced in the intervention period. While the collected information did not allow us to adjust the models directly, it helped us in the interpretation of resulting effect estimates.

For each of the indicators included in our impact evaluation, we specified three different DID models to address research questions 1 to 3. All analyses described below were applied exclusively to primary-level facilities. The twelve secondary-level facilities included in our sample were excluded from analysis due to lack of comparable controls. The panel structure of the data at the facility/village level allowed us to strengthen the precision of our estimations through the inclusion of facility/village fixed effects in the models, thereby controlling for time-invariant unobserved differences across health facilities/villages. Moreover, as an additional robustness test, we also estimated all effects relying only on the partial panel subsamples at the individual and household level.

Given the specific equity focus of the impact evaluation (research question 4), for all indicators relying on household data we additionally performed all analyses with the subsample that contains only units from the lowest socio-economic quintile. This reflects an Intention-to-Treat (ITT) approach, measuring changes at the population level (e.g. “How has health service utilization changed in areas where a targeting of the ultra-poor had taken place, as opposed to areas where only the standard PBF1 was implemented?”) rather than at the individual level (e.g. “How has being selected as indigent changed health service utilization behavior?”). The latter would have been very interesting to explore, but was not possible within the standard HRITF methodology for PBF impact evaluations. Specifically, it would have required a full panel at the household member level and a substantially larger sample size to capture a sufficiently high number of individuals in the demographic groups relevant to the main indicators of interest at baseline who would later be selected as ultra-poor as the project rolled out.

#### DID model specification

First, to answer research question 1, we relied on the quasi-experimental design component and compared all PBF facilities/villages (pooled across all intervention arms) to control facilities/villages, leading to the following regression equation:

$$ {Y}_{dfit}={\alpha}_f+\beta \cdotp {2017}_t+\delta \cdotp \left[ PB{F}_d\ast {2017}_t\right]+\phi \cdotp {X}_{it}+{\epsilon}_{dfit}, $$

where Y_*dfit*_ is the outcome variable for individual *i* from facility/village *f* in district *d* at time *t* with *t = {2014, 2017}*. 2017_t_ is a dummy variable indicating endline observations, thus coefficient *β* gives the time fixed effect. *PBF*_d_ is a dummy variable that equals one for individuals in PBF districts and zero for individuals in control districts. α_*f*_ are facility/village fixed effects, and *X*_*it*_ is a set of additional individual-level covariates as relevant to the respective indicator (i.e. health worker, patient, household member, and/or caregiver characteristics). *ϵ*_*dfit*_ is the error term. Following common practice, standard errors were clustered at the district level, which is the level of treatment assignment for the quasi-experimental component of the study design [47]. The coefficient *δ* gives the DID estimate for the effect of being located in a PBF district when compared to non-PBF districts.

Second, to answer research question 2, we relied on the quasi-experimental design component, but compared single PBF intervention arms to the controls. This leads to the following regression equation:

1 $$ {Y}_{dfit}={\alpha}_f+\beta \cdotp {2017}_t+{\sum}_{k=1}^4{\delta}_k\cdotp \left[ PB{F}_d^k\ast {2017}_t\right]+\phi \cdotp {X}_{it}+{\epsilon}_{dfit}, $$

where $PB{F}_d^k$ is are dummy variables that equal one for individuals from facilities/village in treatment arm PBF_k_, where k = {1,2,3,4}. Individuals from facilities/village in control districts provide the comparison group. The DID estimates *δ*_*k*_ give the effects of PBF_k_ in comparison to status quo (control districts). The remaining equation components are equal to specification 1. Note that as in specification 1, standard errors were clustered at the district level, the level of treatment assignment.

Third, to answer research question 3, we relied on the experimental design component (randomized controlled trial) nested within the quasi-experiment and compared the PBF arms with built-in equity measures (PBF2, PBF3, and PBF4) to the standard PBF (PBF1). For this exercise we had to estimate two separate regression equations; eq. 2b to compare PBF4 to PBF1 in the two districts of Boucle du Mouhoun, where the combination of insurance and PBF and PBF alone had been randomly assigned across facilities, and eq. 2a to compare PBF2 and PBF3 to PBF1 in the eight remaining districts:

2a $$ {\mathrm{Y}}_{fit}={\alpha}_f+\beta \cdotp {2017}_t+{\delta}_2\cdotp \left[T{2}_f\ast {2017}_t\right]+{\delta}_3\cdotp \left[T{3}_f\ast {2017}_t\right]+\phi \cdotp {X}_{it}+{\epsilon}_{fit} $$

2b $$ {\mathrm{Y}}_{fit}={\alpha}_f+\beta \cdotp {2017}_t+{\delta}_4\cdotp \left[T{4}_f\ast {2017}_t\right]+\phi \cdotp {X}_{it}+{\epsilon}_{fit} $$

where Y_*fit*_ is the outcome variable for individual *i* from facility/village *f* at time *t* with *t = {2013, 2017}* in the intervention districts. In this set of analyses, standard errors were clustered at health facility/village level, the level at which random assignment into the four PBF models took place. *δ*_2_ and *δ*_3_ give the DID estimate for the effect of being located in PBF2 compared to PBF1 and PBF3 compared to PBF1, respectively. *δ*_4_ gives the DiD estimate for the effect of being located in PBF4 compared to PBF1.

By design, the quasi-experimental study component is challenged by a relatively low number of clusters (24 districts), as it was not possible to randomize intervention and control facilities at facility level. Too few clusters might lead to the estimation of downward-biased standard errors and, consequently, to an over-rejection of the null hypothesis that there is no program effect. Thus, there is an elevated risk of postulating significant program effects when there is actually no effect detectable with the design. There appears to be no consensus in the literature yet as to which number of clusters is sufficient, but 24 clusters are on the lower end of the spectrum of sufficiency in available simulation studies [62, 63]. Further, studies have shown that the implications of too few clusters are considerably worse when clusters are strongly imbalanced in terms of within-cluster sample sizes as it is unfortunately the case in our study design [64, 65]. The available literature proposes several robustness tests [62, 66]. In a simulation study, Cameron, Gelbach and Miller [63] investigated different recently suggested bootstrapping methods to obtain asymptotic refinement in a scenario with as few as five clusters. They found that the ‘wild bootstrap’ can considerably improve statistical inference of the coefficient estimate and produces much lower over-rejection rates of the H0 than, for instance, the common practice of bootstrapping standard errors. Following this literature, as a robustness test, we applied the ‘wild bootstrap’ to all specification 1 models. In contrast to bootstrapping standard errors, the ‘wild bootstrap’ involves a bootstrap t-procedure [67], where the Wald statistic is bootstrapped, and where the resulting distribution of the Wald statistic is used to confirm or reject inference on the original Wald statistic obtained in the DID regressions.

## Discussion

In this paper, we described the study design and the analytical approach adopted to evaluate a complex health intervention, the health-sector PBF program rolled out since 2014 in Burkina Faso. Our wish has been that of illustrating the way we reconciled scientific and pragmatic concerns to generate policy-relevant evidence on the intervention’s impact, which is methodologically rigorous in its identification strategy but also considerate of the context within which the intervention took place. In particular, we highlighted how we formulated the research questions, ultimately leading to our design choices, on the basis of the knowledge needs expressed by the policy and implementing stakeholders. Moreover, we emphasized how knowledge needs differed across stakeholders and how the wish to accommodate these differences and meet all expectations led us to combine experimental and quasi-experimental methods into a single study design. Since most of the evaluation literature, both methodological and applied, presents one approach at a time (either experimental or quasi-experimental), we trust that our work can be useful for other researchers facing similar challenges when trying to accommodate multiple knowledge needs and to adjust a research design to the realities within which an intervention takes place.

In describing our study design, we have aimed to be very transparent about its methodological weaknesses resulting in particular from the inability to randomize intervention and control within the same districts or enlarge the district sample, the inability to construct panels at the level of unit of observation for most indicators, and potential contamination by various concurrent interventions. Since we have already discussed these challenges and our approaches to addressing them above, we will not reiterate them here. What we wish to highlight here is the fact that all major methodological challenges were beyond our capacity to influence as researchers. In this paper, we showed how we worked ex-post to address them to the best extent possible to ensure maximal accuracy and credibility of our findings.

In conclusion, our experience makes a case for the feasibility of combining quasi-experimental and experimental approaches within a single study design to address multiple knowledge needs and, at the same time, respect local wishes pertaining to implementation practices. However, our experience also shows that compared to a standard randomized trial, such a nested approach is likely to involve certain methodological compromises and implications thereof, which are only partially addressable at the analytical stage. In particular, our lessons learned include the importance of an early and in-depth discussion about the advantages and disadvantages of different design options with all involved stakeholders, to agree on a common set of priorities and streamline expectations; to construct panels if anyhow possible; and to collect additional data and information allowing to test assumptions and deepen contextual understanding.

## Data Availability

Not applicable

## Abbreviations

ANC: Antenatal care
CBHI: Community-based health insurance
CSPS: Centre de Santé et de Promotion Sociale
DID: Difference-in-Differences
GDP: Gross domestic product
HMIS: Health management information system
HRITF: Health Results Innovation Trust Fund
ITT: Intent-to-Treat
MCH: Maternal and Child Health
MoH: Ministry of Health
MRC: Medical Research Council
PBF: Performance-based Financing
U5: Children under the age of five
