# Correction to: Responding to policy makers’ evaluation needs: combining experimental and quasi-experimental approaches to estimate the impact of performance based financing in Burkina Faso

**DOI:** 10.1186/s12913-019-4714-9

**Published:** 2019-11-28

**Authors:** Manuela De Allegri, Julia Lohmann, Aurélia Souares, Michael Hillebrecht, Saidou Hamadou, Hervé Hien, Ousmane Haidara, Paul Jacob Robyn

**Affiliations:** 1 Institute of Global Health, Medical Faculty, Heidelberg University, Germany, Im Neuenheimer Feld 130.3, 69120 Heidelberg, Germany; 2 The World Bank, Nouvelle Route Bastos B, P 1128 Yaoundé, Cameroon; 3 Centre MURAZ, 2054 Avenue Mamadou KONATE, 01 B.P. 390, Bobo-Dioulasso 01, Burkina Faso; 4 The World Bank; Health, Nutrition, Population Global Practice, 1818 H Street, NW, Washington, DC 20433 USA

**Correction to: BMC Health Serv Res**

**https://doi.org/10.1186/s12913-019-4558-3**

Due to an error introduced during typesetting of this article [1], there are two corrections about the figures. 1. The caption of Fig. 1 should be changed to “Study design”. 2. The Fig. 2 is missing.

**Fig. 1:**
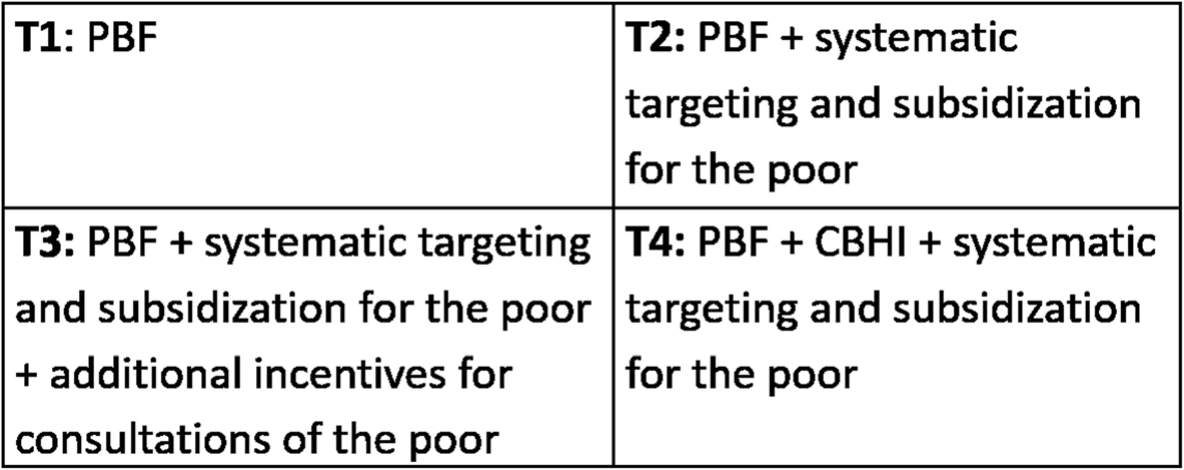
Study design

**Fig. 2:**
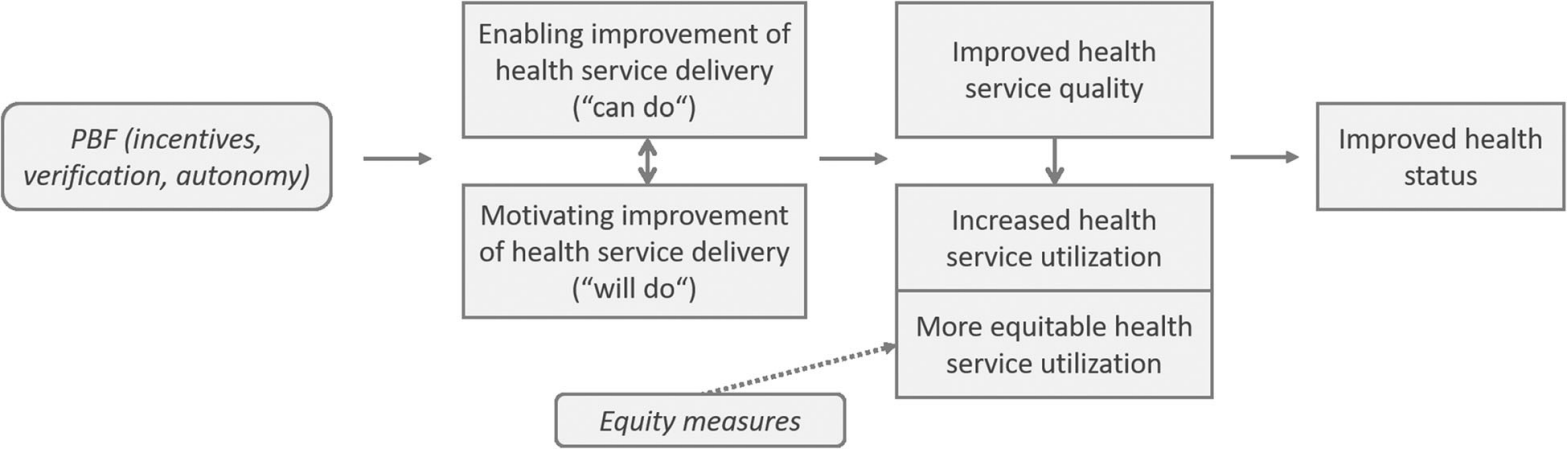
PBF theory of change

The original article has been corrected.
